# Citrus Pests and Diseases Recognition Model Using Weakly Dense Connected Convolution Network

**DOI:** 10.3390/s19143195

**Published:** 2019-07-19

**Authors:** Shuli Xing, Marely Lee, Keun-kwang Lee

**Affiliations:** 1 Center for Advanced Image and Information Technology, School of Electronics & Information Engineering, Chon Buk National University, Jeonju, Chon Buk 54896, Korea; 2 Department of Beauty Arts, Koguryeo College, Naju 520-930, Korea

**Keywords:** citrus, pests and diseases identification, convolutional neural network, parameter efficiency

## Abstract

Pests and diseases can cause severe damage to citrus fruits. Farmers used to rely on experienced experts to recognize them, which is a time consuming and costly process. With the popularity of image sensors and the development of computer vision technology, using convolutional neural network (CNN) models to identify pests and diseases has become a recent trend in the field of agriculture. However, many researchers refer to pre-trained models of ImageNet to execute different recognition tasks without considering their own dataset scale, resulting in a waste of computational resources. In this paper, a simple but effective CNN model was developed based on our image dataset. The proposed network was designed from the aspect of parameter efficiency. To achieve this goal, the complexity of cross-channel operation was increased and the frequency of feature reuse was adapted to network depth. Experiment results showed that Weakly DenseNet-16 got the highest classification accuracy with fewer parameters. Because this network is lightweight, it can be used in mobile devices.

## 1. Introduction

Pests and diseases are the two most important factors affecting citrus yields. Types of citrus pests and diseases are numerous in nature. Some of them are similar in appearance, making it difficult for farmers to precisely recognize them in time. In recent years, developments of convolutional neural networks (CNNs) have dramatically improved the state-of-the-art in computer vision. These new structures of network have enabled researchers to obtain high accuracy for image classification, object detection, and semantic segmentation [1]. Therefore, some studies have adopted the CNN model to identify the category of pests or diseases based on image. Liang et al. [2] have proposed a novel network consisted of residual structure and shuffle units for plant diseases diagnosis and severity estimation. Cheng et al. [3] have compared the classification performance of different depths of CNN models for 10 classes of crop pests with complex shooting background. The highest classification accuracy in both studies was obtained with the deepest network. For detection tasks, people are also more willing to select a very deep network architecture instead of a shallow one. Shen et al. [4] have applied a faster R-CNN [5] framework with improved Inception-V3 [6] to detect stored-grain insects under field condition with impurities. The same feature extractor network and SSD [7] model have been utilized by Zhuang et al. [8] to evaluate the health status of farm broilers.

In theory, the complexity of the CNN model depends on the scale of dataset. However, deep convolutional networks mentioned above were all over-fitted because they were proposed based on ImageNet [9] initially. Although a fine-tuned method [10] can be used to reduce the divergence between training and testing, the space required for model storage is so large that they cannot be deployed on mobile devices with little memory.

In this paper, a simple but effective network architecture was developed to classify pictures of citrus pests and diseases. Our network design principles focused on improving the utilization of model parameters. There has been evidence suggesting that some feature maps generated by convolutions are not useful [11,12]. To decrease the impact of redundant features on classification, Hu et al. [13] and Woo et al. [14] have introduced an attention mechanism to suppress unnecessary channels. Their approaches are more adaptable than the Dropout [15] and stochastic depth [16]. However, the extra branch in each building block increases the overhead of a network. Unlike these approaches, the channel selection of this paper was implemented through the method of cross-channel feature fusion. In Network in Network [17], two consecutive 1 × 1 convolutional layers were regarded as a way to enhance model discriminability for local patches. From another perspective, this structure is also a good choice to refine feature maps. Highway network [18] first provided the idea of feature reuse to ease the optimization difficulty suffered by deep networks. ResNet [19] generalized it with identity mappings. DenseNet [20] further boosted the frequency of skip-connection. DenseNet has a better representation ability than ResNet because it can produce a higher accuracy with fewer parameters. The concatenation operation of DenseNet was followed but some connections between long-range layers were removed by us. Because of this weakly dense pattern, our network is called Weakly DenseNet.

Experiment results showed that Weakly DenseNet achieved the highest accuracy in classifying citrus pests and diseases. With regard to computational complexity, our proposed model is also lightweight. These phenomena indicate that the optimization of network structure is more important than blindly increasing the depth or width. The main contributions of this work are summarized as follows:

A specific image dataset of citrus pests and diseases is created. It is a relatively complete image dataset for the diagnosis of citrus pests and diseases.

A novel and lightweight convolutional neural network model is proposed to recognize the types of citrus pests and diseases. The network design is based on improving parameter efficiency.

A new data augmentation method is developed to reinforce model generalization ability, which can significantly reduce the similarity between generated images.

## 2. Related Work

Pests and diseases can cause great damage to crops if they are not controlled. To recognize them, farmers used to rely on experienced experts. With the popularity of image sensors, using computer vision methods to identify pests and diseases has become a trend. Boniecki et al. [21] have proposed to use image analysis techniques and artificial neural network model to classify images of apple pests in simple background. Their dataset included 12,000 images from six species of apple pests which are most commonly found in Polish orchards. For training and testing proposed artificial neural network model, seven selected coefficients of shape and 16 color characteristics were extracted from each pest image as inputs. Sun et al. [22] have combined SLIC (simple linear iterative cluster) with SVM (support vector machine) classifier to detect diseases on tea plant. Their algorithm improved the prediction accuracy of disease images taken with complex backgrounds but needed more pre-treatments to reduce interference. A total of 1308 pictures from five common tea plant diseases were included in their dataset. These images were divided into two parts with a ratio of 4:1 for training and testing. Ferentinos [23] has employed deep CNN models to perform plant disease detection and diagnosis. They used an open database which contains 87,848 photographs of leaves to train each model. Images without pre-processing were regarded as inputs in his study. Compared with other selected models, VGG achieved the highest success rate with 99.48%. These advantages of deep CNNs have encouraged more researchers to apply them in the agricultural field.

A wide range of CNN architectures has been proposed to improve performance. VGGNets [24] first use small size convolution filters to reduce parameters and increase depth. ResNet exploits a simple identity skip-connection to ease optimization issues of deep networks. WideResNet [25] replaces the bottleneck structure in ResNet with two broad 3 × 3 convolutional layers to reduce depth. DenseNet enhances deep supervision [26] by iteratively concatenating input features with output features. Xception [27] introduces a depthwise separable convolution to decrease the number of parameters in a regular convolution. In it, depthwise convolution is responsible for feature extraction and pointwise convolution (a regular 1 × 1 convolution) is used for cross-channel feature fusion. This new convolution operation has become a core component of many lightweight networks, such as MobileNets [28,29] and ShuffleNets [30,31]. The structure of MobileNet-v1 is similar to that of VGG. MobileNet-v2 develops an inverted residual block to increase memory efficiency. To maintain the representational power of narrow layer in each inverted residual block, ReLU activation [32] behind it is removed. ShuffleNet-v1 employs group convolution [33] to further reduce the computational cost of depthwise separable convolution, a channel shuffle operation is adopted to enhance the information exchange of subgroups. ShuffleNet-v2 is constructed based on ShuffleNet-v1. However, it suggests splitting channels into two equal parts and using concatenation instead of addition to execute feature reuse. People tend to use their architectures designed for the ImageNet without considering their own dataset scale. This behavior may lead to overfitting problems and waste of computing resources. Different from previous approaches, a novel, and lightweight network was constructed to classify images in our dataset.

## 3. Dataset

The dataset used in our experiment included 17 species of citrus pests and seven types of citrus diseases. Pests’ images were mainly collected from the Internet. Images of diseases were taken in a tangerine orchard of Jeju Island using a high-resolution camera. Our image dataset is available at the website of Appendix B. Table 1 shows the name and number of images of each kind of pest and disease.

### 3.1. Image Collection of Citrus Pests

**Figure 1:**
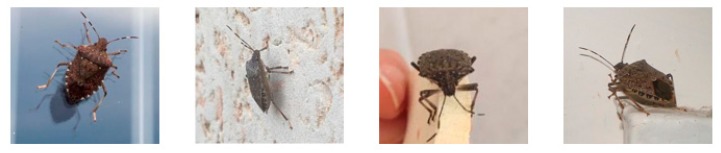
Pictures of brown marmorated stink bug taken from different angles.

**Figure 2:**
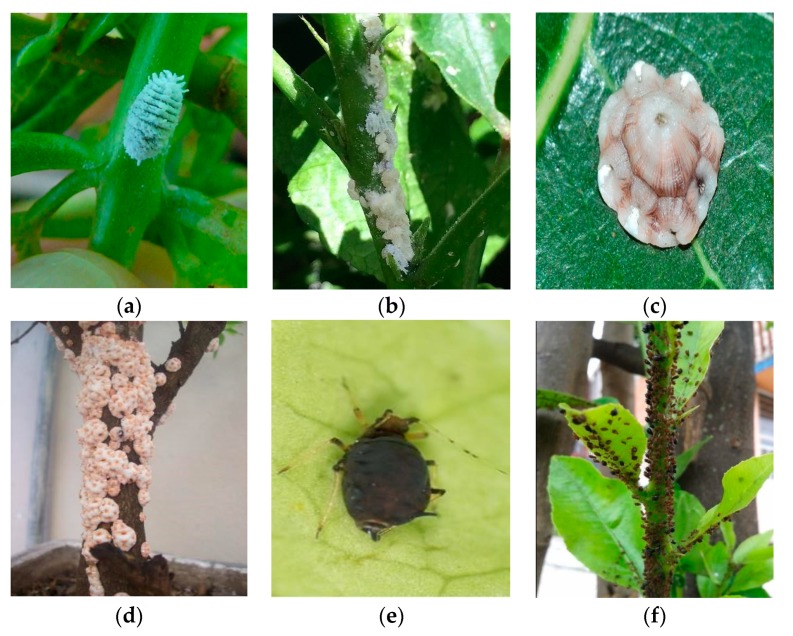
Examples of small size citrus pests: (**a**), (**c**), and (**e**) are the images of their individuals, (**b**), (**d**), and (**f**) are those of their groups.

Insect pests have metamorphosis properties. We focused on images of adults. This is because other stages in their life cycles are short and rare to observe. The appearance of the same pest can vary significantly from one viewing angle to another (refer to Figure 1). To reduce the effect of shooting angle on classification accuracy, photos of pests taken from different angles were gathered. Some citrus pests have small sizes, such as aphid, mealybug, and scale. It is difficult to capture images of their individuals and most of them live by groups to resist predators. For these species, pictures of their group living on a tree were collected (refer to Figure 2).

### 3.2. Image Collection of Citrus Diseases

**Figure 3:**
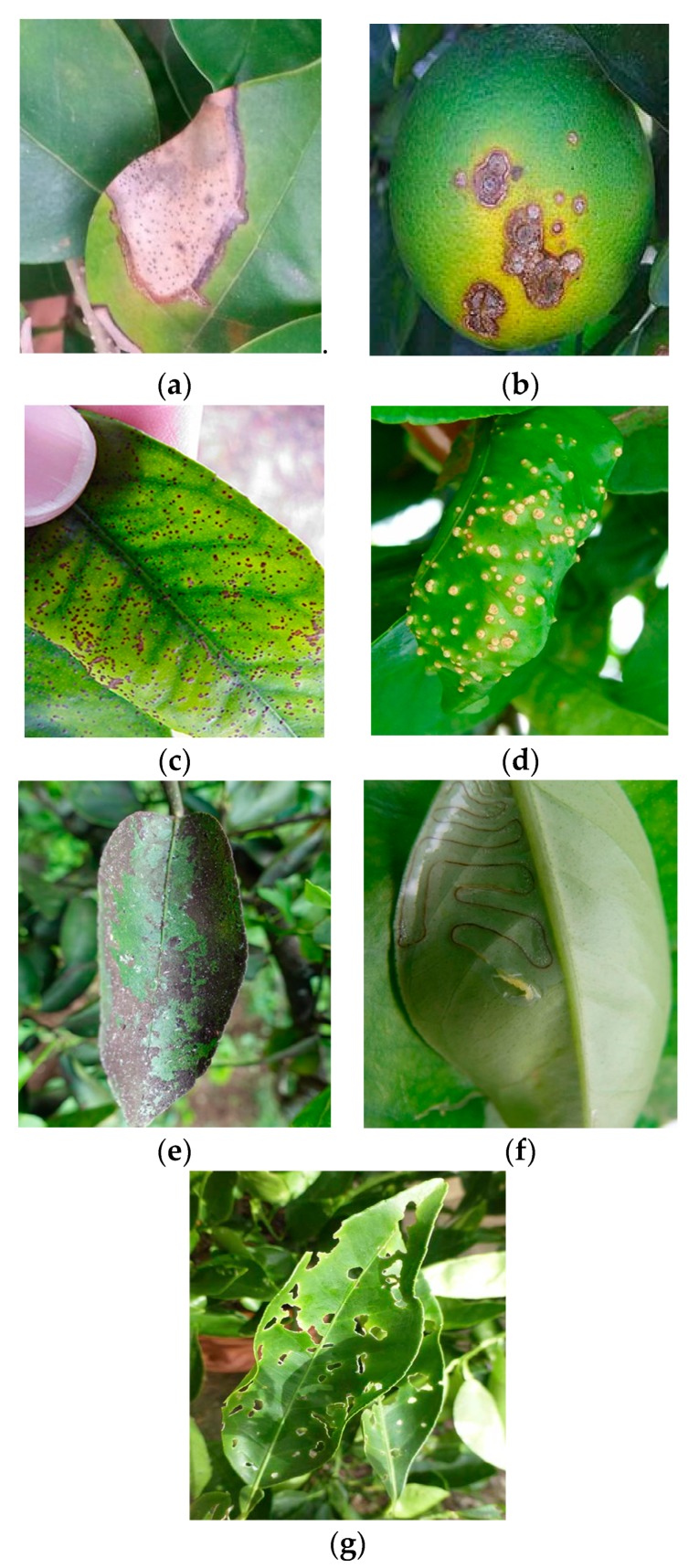
Representative images of citrus diseases. (**a**) citrus anthracnose, (**b**) citrus canker, (**c**) citrus melanose, (**d**) citrus scab, (**e**) sooty mold, (**f**) leaf miner, and (**g**) pest holes.

Compared with pests, features of citrus diseases are more regular. Pictures of citrus diseases were mainly taken in the summer after a heavy rain because the incidence was higher than usual. To keep more details, the distance between camera and diseases was close. Some diseases will cause leaf holes at a later phase. To enhance comparison, images of the leaf holes created by pests (PH) were included as a disease label. Figure 3 displays sample images of each disease.

### 3.3. Data Augmentation

**Figure 4:**
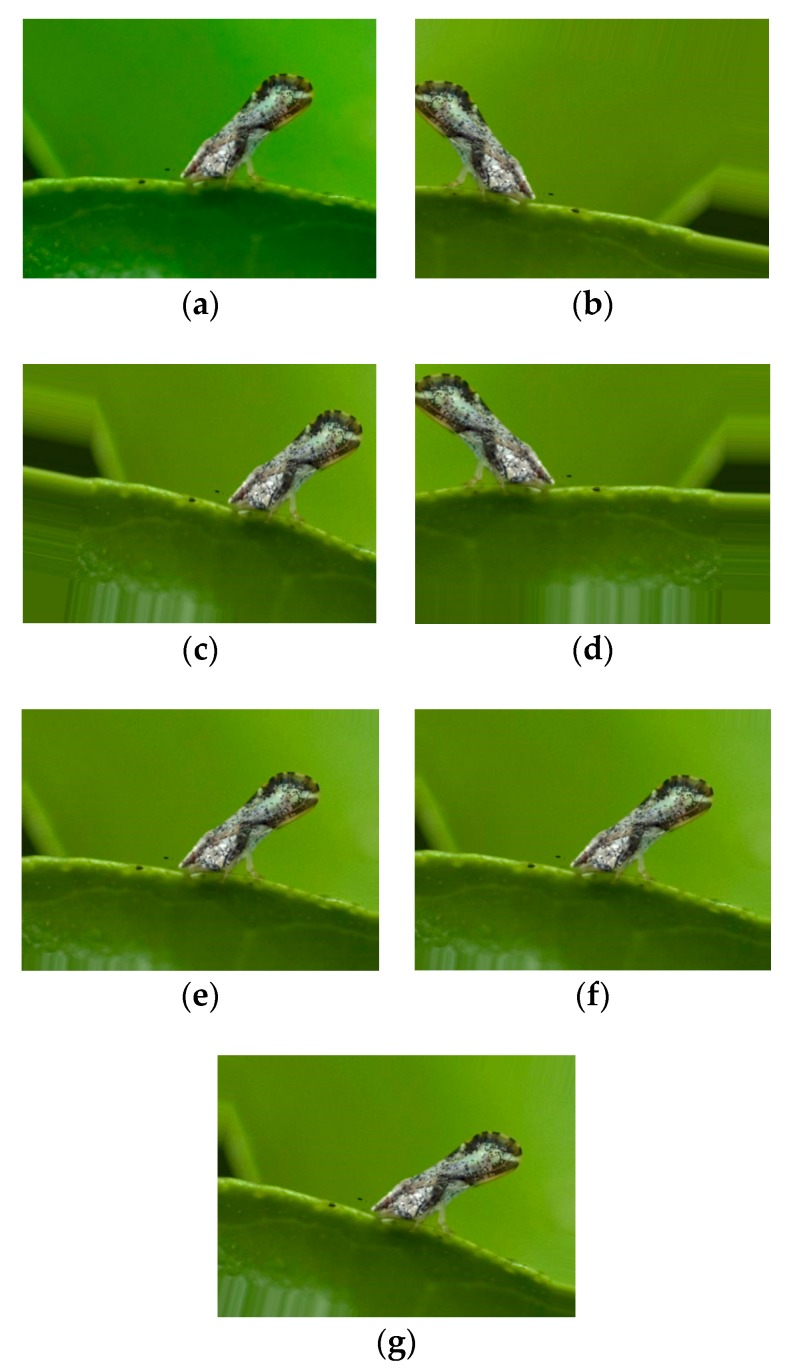
Comparison between different data augmentation methods. (**a**) Original image, (**b**), (**c**), and (**d**) images generated from proposed algorithm, (**e**), (**f**), and (**g**) pictures produced by a single rotation operation.

The problem of imbalanced data classification has been discussed by Das et al. [34]. It prompted us to increase the number of images in the class whose dataset scale was smaller than that of others. For augmenting image data, the generic practice is to perform geometric transformations, such as rotation, reflection, shift, and flip. However, images generated by a single type of operation are similar to each other. They increase the probability of overfitting. To avoid this situation, a new data augmentation method was proposed, which could randomly select three kinds of operations and combine them together to produce new images. Available operations and values of them are shown in Table 2. Figure 4 presents pictures obtained from this approach.

## 4. Weakly DenseNet Architecture

Convolutional layers in the CNN model are responsible for feature extraction and generation. Therefore, many researchers have focused on increasing depth and width to improve classification accuracy. In contrast, the proposed Weakly DenseNet was created to improve parameters’ utilization. To reach this goal, a complex cross-channel operation was adopted to refine feature maps and concatenation method was used for feature reuse.

### 4.1. The 1 × 1 Convolution for Feature Refinement

A regular convolution contains two aspects: Local receptive field and weight share. From the local receptive field point of view, a 1 × 1 convolution regards each pixel of a feature map as input. However, when weight share is considered, it was equivalent to the whole feature map multiplied by a learnable weight. Therefore, this kind of convolution has the function of refining feature maps.

**Figure 5:**
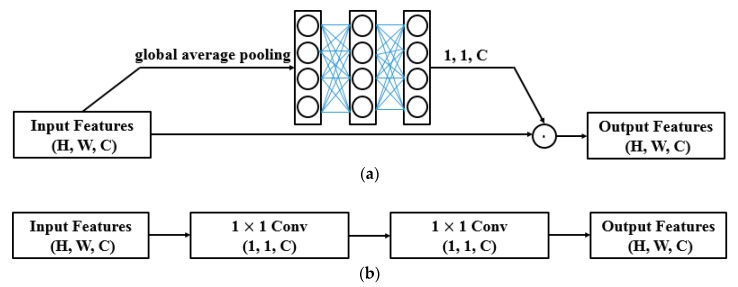
Feature refinement. (**a**) Squeeze-and-excitation block, (**b**) the proposed method.

One layer of 1 × 1 convolution only implements a linear transformation. Many network architectures just use it to alter channel dimension [19,20]. To extend the functionality of 1 × 1 convolution, two layers of it were stacked after each 3 × 3 convolutional layer. The proposed structure takes each whole feature map as an input and thus does not need an extra branch to execute feature recalibration. This reduces the optimization difficulty in contrast with SENet [13]. Figure 5 illustrates the difference between them.

### 4.2. Feature Reuse

As network depth increases, gradient propagation becomes more difficult. ResNet addresses this issue by adding input features to output features across a few layers (1). DenseNet simplifies the addition operation by concatenation, which allows feature maps from different depths to combine along channel dimension (2). Considering operation efficiency, the concatenation method of DenseNet was selected for feature reuse

(1) $$\tilde{X}=X+H(X)$$

(2) $$\tilde{X}=\{X,H(X)\}$$

where *X* represents inputs, *H(X)* is defined as the underlying mapping, $\tilde{X}$ denotes outputs. In the addition operation, *X* and $\tilde{X}$ should have the same dimension.

However, overuse of features of previous layers can increase network overhead. To solve this problem, DenseNet employs a transition layer to reduce the number of input features for a dense block. The dense connectivity pattern of DenseNet also made low-level features to be repeatedly used many times. Yosinski et al. [35] have proved that features generated by convolutions far from the classification layer are general, thus contributing little to classification accuracy. According to their conclusion, some connections between long-range layers were removed.

### 4.3. Network Architecture

**Figure 6:**
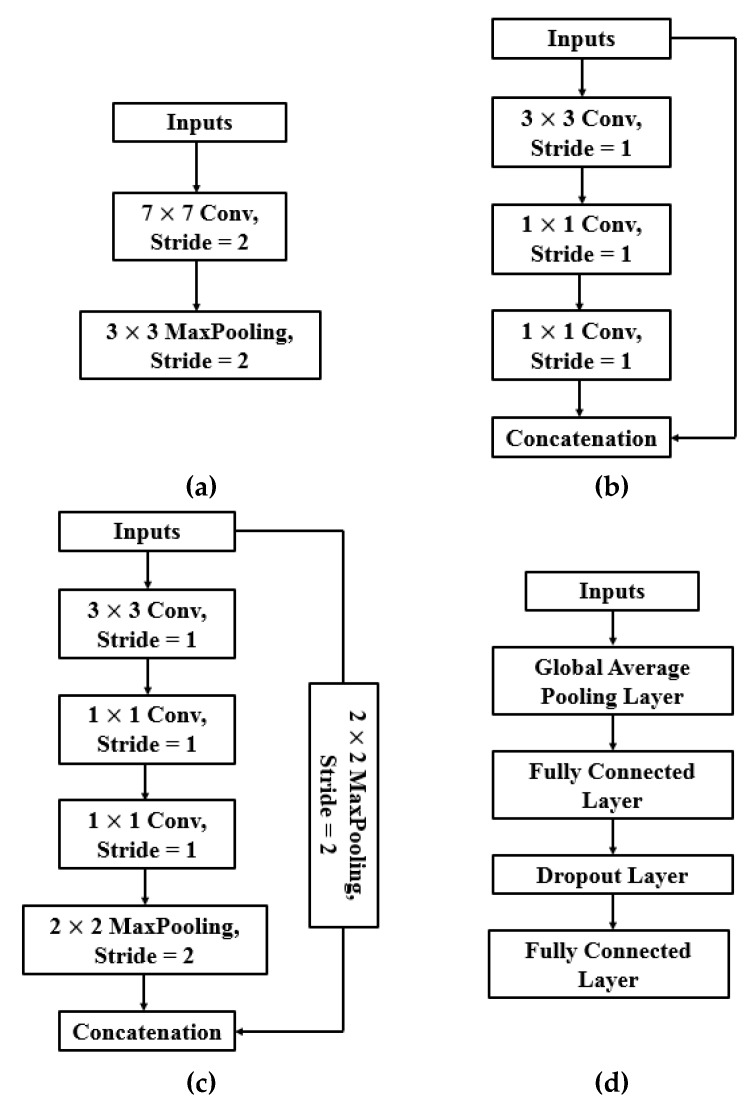
Building blocks of the Weakly DenseNet. (**a**) initial building block, (**b**) and (**c**) intermediate building blocks, (**d**) final classification building block.

The network architecture was divided into three parts during design. Figure 6 demonstrates the building block of each part. As for the frequency (*v*) of feature reuse, it was adapted to the depth of the network:

Features generated by adjacent convolutions are highly correlated [6]. A final classification layer concentrates on using high-level features [20]. Therefore, keeping connections between short-distance layers and reducing the combination of low-level features to the classification layer are necessary.

**Figure 7:**
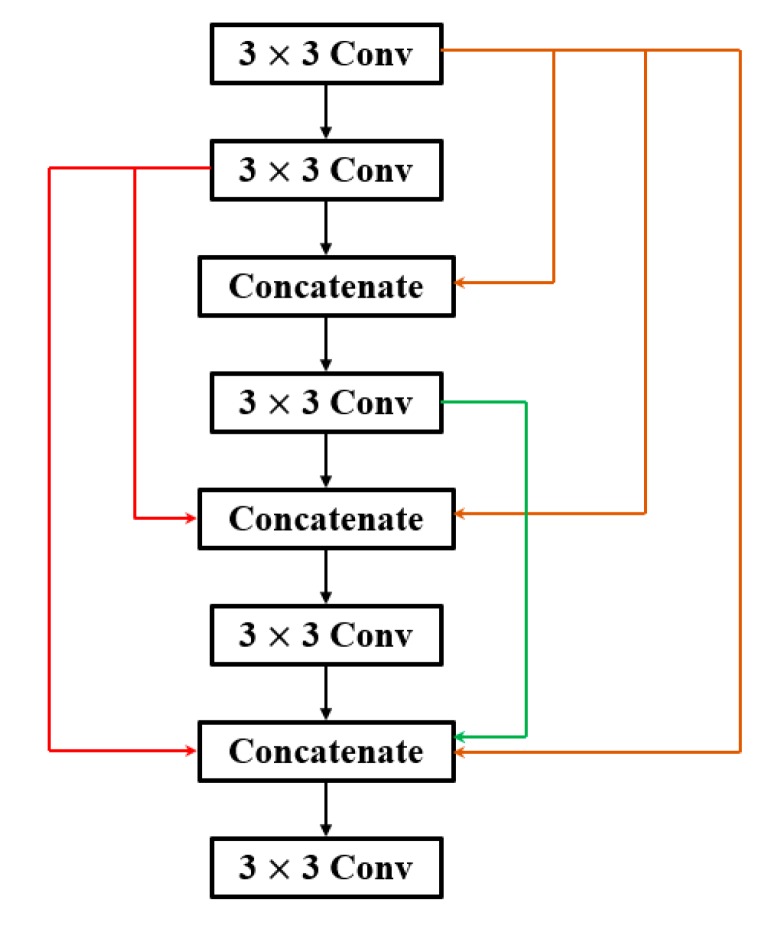
Building block of DenseNet.

Middle layers produce features are between general and specific [35]. It is assumed that if the network depth is increased, the value of *v* in the middle layers should be enlarged. Figure 7 illustrates the building block of DenseNet for fitting ImageNet dataset. In it, *v* > 1.

We set *v* to 1, because our network was constructed not very deeply based on image data scale of citrus pests and diseases. Table 3 summarizes the architecture of the network.

Each convolution in the building block is followed by a batch normalization layer [36] and a ReLU layer [32].

## 5. Experiments and Results

The original dataset was split into three parts: Training set, validation set, and test set. The ratio between them was 4:1:1. The images in each set were resized to 224 × 224 by a bilinear interpolation approach. To evaluate the effectiveness of Weakly DenseNet, it was compared with several baseline networks from different aspects. The software implementation was based on Keras with TensorFlow backend. The hardware foundation was GPU, 1080Ti. Code and models are provided in Appendix C.

### 5.1. Training

All the networks were trained with SGD and a Nesterov momentum [37] of 0.9 was introduced to accelerate convergence. To improve models’ generalization performance, a small batch size of 16 was selected during training [38]. The initial learning rate was set to 0.001 and it can be adjusted based on Algorithm 1.

**Algorithm 1.** Learning Rate Schedule**Input:** Patience *P*, decay θ, validation loss *L* **Output:** Learning rate γ 1: Initialize *L* = *L*_0_, γ$\;=\;\gamma^{0}$ 2: *i* ← 0 3: **while**
*i* < *P*
**do** 4:  **if**
*L*
≤
*L_i_*
**then** 5:   *i* = *i* + 1 6:  **else** 7:   *L* = *L_i_* 8:   *i* = *i* + 1 9:  **end if** 10: **end while** 11: **if**
*L* = *L*_0_
**then** 12:   γ = γ * θ 13: **end if**


In the experiment, *P* = 5 and *θ* = 0.8. Weight initialization proposed by He et al. [39] was followed, and a weight decay of $10^{-4}$ was used to alleviate the overfitting problem. The maximum training epoch of each model was 300. The rate of Dropout was set to be 0.5.

The VGG-16 of this paper used a global average pooling layer [17] to reduce the number of parameters in fully connected layers. In the original SE block, the size of the hidden layer was reduced by a ratio (*r* > 1) to limit model complexity. To keep the same computation cost as NIN-16, the value of *r* was set to 1 by us.

**Figure 8:**
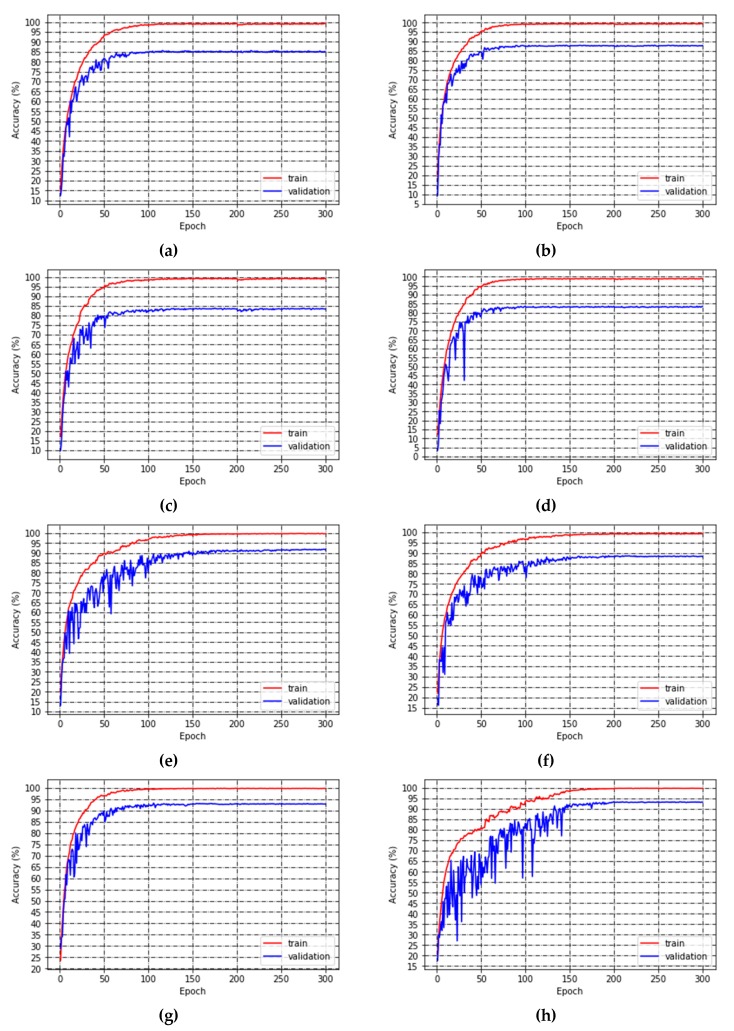
Training plot of each model. (**a**) MobileNet-v1, (**b**) MobileNet-v2, (**c**) ShuffleNet-v1, (**d**) ShuffleNet-v2, (**e**) NIN-16, (**f**) SENet-16, (**g**) VGG-16, (**h**) WeaklyDenseNet-16.

Table 4 shows the training results of each model. With regard to classification accuracy, WeaklyDenseNet-16 was the highest, followed by VGG-16, and NIN-16. The higher accuracy of NIN-16 than SENet-16 indicates that two layers of 1 × 1 convolution have better performance of refining feature maps than SE block. By concatenating previous layers’ features, the recognition performance of NIN-16 was significantly strengthened: The accuracy of WeaklyDenseNet-16 was 1.58 percent higher than that of NIN-16. As for computation cost, VGG-16 was the largest while that of SENet-16 was the least. It should be noticed that ShuffleNets and MobileNets overfitted citrus pests and diseases image dataset. Even though they had similar model sizes to WeaklyDenseNet-16, their larger values of depth brought bigger error rate on validation dataset. As for training speed per batch size, bigger size model took more time except for ShuffleNet-v2 [31]. The accuracy training plots of benchmark models are displayed in Figure 8. It can be seen that each model completely converges in 300 epochs.

### 5.2. Test

**Figure 9:**
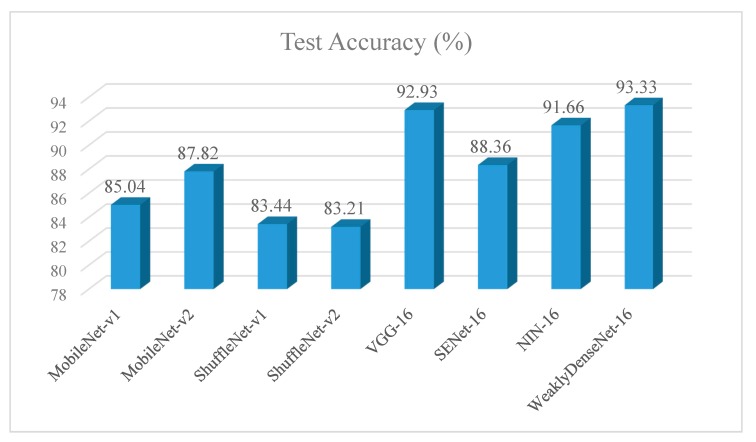
Comparison of the test accuracy.

**Figure A1:**
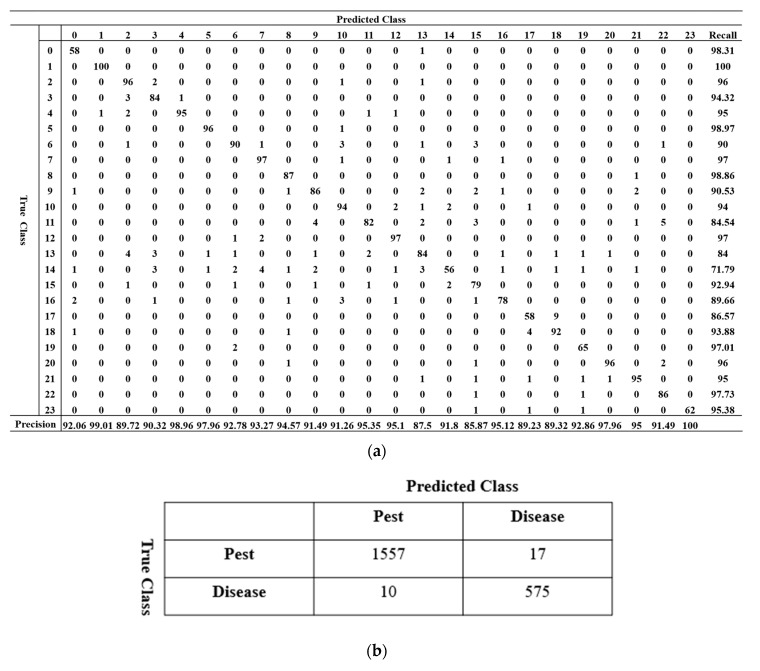
The confusion matrix for the test set. (**a**) test result of each class, (**b**) prediction for pest and disease label.

Test accuracy results of selected models are shown in Figure 9 which shows the same accuracy trend as Table 4. The confusion matrix of Weakly DenseNet-16 on the test dataset is presented in Figure A1 (refer to Appendix A). Figure A1a shows that the recall rate (3) of citrus root weevil is the lowest and that of the citrus swallowtail is the highest. Among misclassified images, citrus anthracnose and citrus canker are the most easily confused by the proposed model: Nine images of citrus anthracnose were considered as the class of citrus canker and four images of citrus canker were regarded as citrus anthracnose by the network. The two diseases at later phase show a similar appearance on leaves. Thus, Weakly DenseNet-16 gave some incorrect predictions. The precision rate (4) of the PH is the largest while that for citrus flatid planthopper is the lowest. Figure A1b displays the wrong predictions between citrus pest and disease. Ten images of citrus disease were misclassified into pest labels and seventeen pictures of citrus pest were identified as diseases by mistake. In them, the probability that citrus soft scale is falsely regarded as citrus sooty mold is the highest. Adult citrus soft scales can secrete honeydew sooty mold around them for growth, which shows a similar pattern to the symptom of sooty mold.

(3) $$\mathrm{Recall}\;=\;\frac{Number\;of\;true\;positive\;samples}{Number\;of\;true\;positive\;samples\;+\;Number\;of\;false\;negative\;samples}\times 100\%$$

(4) $$\mathrm{Precision}\;=\;\frac{\mathrm{Number}\;\mathrm{of}\;\mathrm{true}\;\mathrm{positive}\;\mathrm{samples}}{\mathrm{Number}\;\mathrm{of}\;\mathrm{true}\;\mathrm{positive}\;\mathrm{samples}\;+\;\mathrm{Number}\;\mathrm{of}\;\mathrm{false}\;\mathrm{positive}\;\mathrm{samples}}\times 100\%$$

**Figure 10:**
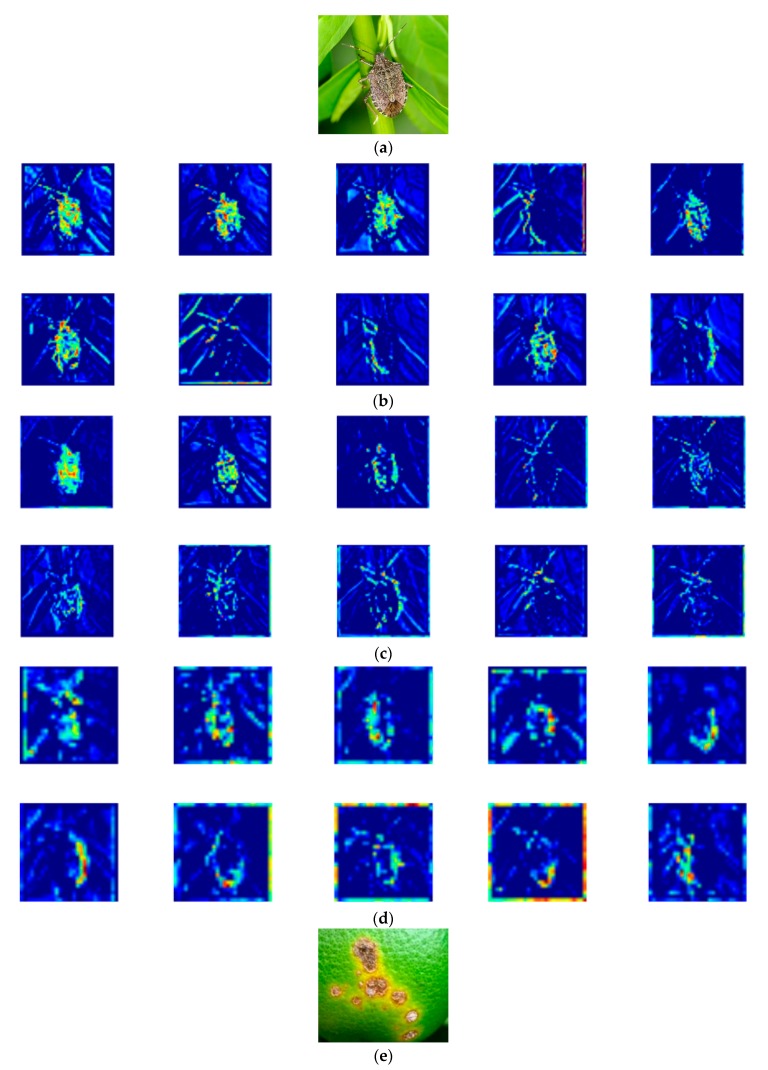

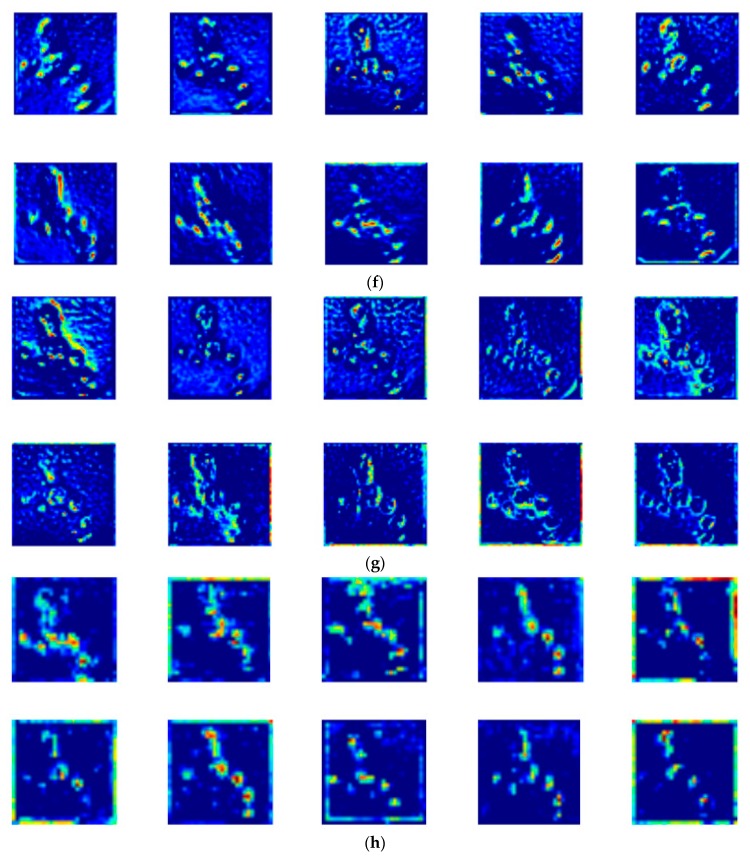
Visualization of features. (**a**) and (**e**) input images, (**b**) and (**f**) output features of the intermediate building block 1, (**c**) and (**g**) sampled features of the intermediate building block 2, (**d**) and (**h**) examples of the feature maps in the intermediate building block 3. Brighter color in images corresponds to higher value.

The hierarchical structure of the CNN model allows features generated from layers of different depths to show significant differences [40]. To better understand the learning capacity of intermediate building blocks of Weakly DenseNet-16, several important feature maps of them were visualized and compared. From Figure 10, it can be noticed that:

A bank of convolutional filters in the same layer can extract features of different parts of the target object. This feature extraction method allows the CNN model to acquire sufficient visual information for subsequent analysis.

With increasing depth, the background features become less visible. Therefore, CNN models do not require additional pre-processing techniques to reduce background noise. They are more convenient to use than conventional machine learning algorithms.

Features of the deeper layer are more abstract than those of the shallow layer. More convolution and max pooling operations are performed on shallow layer features in the deeper layer, resulting in higher-level features that are more suitable for classification.

## 6. Conclusions and Future Work

Pests and diseases can reduce citrus output. To control their impact, a new image dataset about citrus pests and diseases was created and a novel CNN architecture was proposed to recognize them. The network was constructed from the aspect of improving parameters’ utilization instead of depth and width. The structure of two 1 × 1 convolutional layers was revisited and applied to refine feature maps. To relieve the optimization difficulty in the deep network, the idea of feature reuse was followed. Considering operation efficiency, the concatenation method of DenseNet was employed. However, the high frequency of feature reuse increased the overhead of network. To save computation cost, the value of feature reuse frequency was set based on network depth. To further improve the robustness of the CNN model, a new data augmentation algorithm was provided, which can significantly lessen the similarity between generated images. In experimental studies, NIN-16 got a test accuracy of 91.66% which was much higher than that of SENet-16 (88.36%). This phenomenon indicates that two-layer 1 × 1 convolution has better performance of refining feature maps than SE block. The higher accuracy of WeaklyDenseNet-16 (93.33%) than NIN-16 indicates that feature reuse method can further enhance network performance. VGG-16 achieved the second-highest classification accuracy (93%) but consumed the most computing resources: Model size is 120.2 MB. This fact implies the importance of network structure optimization on fitting different datasets.

The object scale in the image is an essential factor that influences classification accuracy of the CNN model. Using extremely deep networks to identify big scale objects will cause a waste of computational resources. For the identification of small size objects, shallow networks cannot give accurate results. Future work is to build a CNN model that can adapt to the size of the object in the image.

## Figures and Tables

**Table 1:** The description of citrus pests and diseases image dataset.

Class ID	Common Name	Scientific Name	Number of Samples
	**Citrus Pests**		
8	Mediterranean fruit fly	Ceratitis capitata	558
0	Asian citrus psyllid	Diaphorina citri Kuwayama	359
5	Citrus longicorn beetle	Anoplophora chinensis	597
7	Brown marmorated stink bug	Halyomorpha halys	606
3	Southern green stink bug	Nezara viridula	488
4	Fruit sucking moth	Othreis fullonica	600
1	Citrus swallowtail	Papilio demodocus	600
15	Citrus flatid planthopper	Metcalfa pruinosa (Say)	555
9	Citrus mealybug	Planococcus citri	495
13	Aphids	Toxoptera citricida	514
11	Citrus soft scale	Hemiptera: Coccidae	497
12	False codling moth	Thaumatotibia leucotreta	511
14	Root weevil	Diaprepes abbreviatus, Pachnaeus opalus	378
2	Forktailed bush katydid	Scudderia furcata	600
10	Cicada	Cicadoidea	508
6	Garden snail	Cornu aspersum	618
16	Glassy-winged sharpshooter	Homalodisca vitripennis	567
	Total		9051
	**Citrus Diseases**		
17	Anthracnose	Colletotrichum gloeosporioides	467
18	Canker	Xanthomonas axonopodis	598
20	Melanose	Diaporthe citri	532
21	Scab	Elsinoë fawcettii	503
19	Leaf miner	Liriomyza brassicae	427
22	Sooty mold	Capnodium spp	568
23	Pest hole		415
	Total		3510

**Table 2:** Parameter set for data augmentation.

Operation	Value
Rotation	[$0^{{}^{\circ}}$, $15^{{}^{\circ}}$]
Width shift	[0, 0.2]
Height shift	[0, 0.2]
Shear	[0, 0.2]
Zoom	[0.8, 1.2]
Horizontal flip	-

**Table 3:** Network architecture for citrus pests and diseases.

Block	Output Size
Initial Block (a)	56 × 56 × 32
Intermediate Block (b)	56 × 56 × 96
Intermediate Block (c)	28 × 28 × 192
Intermediate Block (b)	28 × 28 × 384
Intermediate Block (c)	14 × 14 × 768
1 × 1 conv, stride 1	14 × 14 × 512
1 × 1 conv, stride 1	14 × 14 × 512
2 × 2 max pool, stride 2	7 × 7 × 512
Classification Block (d)	1 × 1 × 24

**Table 4:** Training performance of selected models.

Model Name	Training Accuracy	Validation Accuracy	Model Size (MB)	Training Time (ms)/Batch Size
MobileNet-v1	99.23	85.45	25	152
MobileNet-v2	99.28	87.97	33.9	198
ShuffleNet-v1	99.13	83.58	28.8	145
ShuffleNet-v2	98.72	83.58	42	144
VGG-16	99.82	93	120.2	303
SENet-16	99.10	88.71	19.5	138
NIN-16	99.63	91.84	19.6	137
WeaklyDenseNet-16	99.83	93.42	30.5	138

‘NIN’ represents Network in Network.

## Appendix B

Image dataset is available at: https://files.mycloud.com/home.php?brand=webfiles#23a3c71/device_30757105/ARlab/xingshuli600/.

## Appendix C

Models and code are available at: https://github.com/xingshulicc/xingshulicc/tree/master/citrus_pest_diseases_recognition.
